# (*S*)-5-Hexyl-1-[(*S*)-2-hydr­oxy-1-phenyl­ethyl]-4-meth­oxy-1*H*-pyrrol-2(5*H*)-one

**DOI:** 10.1107/S1600536809014160

**Published:** 2009-04-22

**Authors:** Jian-Feng Zheng, Li-Jiao Jiang, Jian-Nan Guo

**Affiliations:** a Department of Chemistry and the Key Laboratory for Chemical Biology of Fujian Province, College of Chemistry and Chemical Engineering, Xiamen University, Xiamen, Fujian 361005, People’s Republic of China

## Abstract

The title compound, C_19_H_27_NO_3_, was obtained by the reaction of (3*S*,7a*R*)-7a-hexyl-7-meth­oxy-3-phenyl-2,3-dihydro­pyrrolo[2,1-*b*]oxazol-5(7a*H*)-one and triethyl­silane using titanium(IV) chloride as catalyst. In the mol­ecule, the phenyl and dihydro­pyrrolone rings form a dihedral angle of 83.8 (1)°. O—H⋯O hydrogen-bonding inter­actions lead to the formation of a chain parallel to the *a* axis.

## Related literature

For the bioactivity of methyl tetramates, see: Royles (1995 ▶). For the synthesis, see: Jiang *et al.* (2009 ▶).
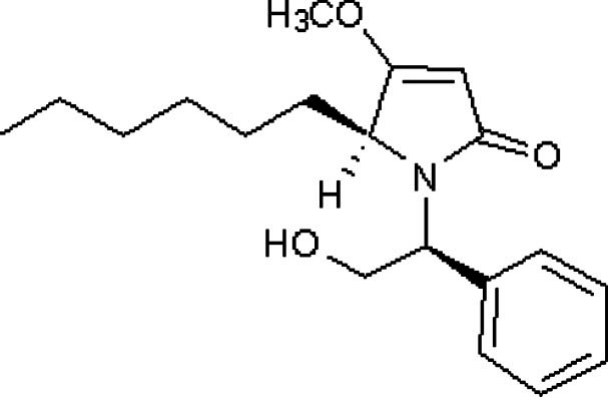


## Experimental

### 

#### Crystal data

C_19_H_27_NO_3_
*M* *_r_* = 317.42
Orthorhombic, 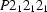

*a* = 9.6739 (17) Å
*b* = 10.0995 (18) Å
*c* = 17.929 (3) Å
*V* = 1751.7 (5) Å^3^
*Z* = 4
Mo *K*α radiation
μ = 0.08 mm^−1^
*T* = 173 K
0.56 × 0.32 × 0.23 mm

#### Data collection

Bruker APEX CCD diffractometer
Absorption correction: multi-scan (*SADABS*; Bruker, 2001 ▶) *T*_min_ = 0.956, *T*_max_ = 0.982
12545 measured reflections
1773 independent reflections
1732 reflections with *I* > 2σ(*I*)
*R*_int_ = 0.023

#### Refinement

*R*[*F* ^2^ > 2σ(*F* ^2^)] = 0.029
*wR*(*F* ^2^) = 0.079
*S* = 1.13
1773 reflections
208 parameters
H-atom parameters constrained
Δρ_max_ = 0.13 e Å^−3^
Δρ_min_ = −0.13 e Å^−3^

### 

Data collection: *SMART* (Bruker, 2001 ▶); cell refinement: *SMART*; data reduction: *SAINT* (Bruker, 2001 ▶); program(s) used to solve structure: *SHELXS97* (Sheldrick, 2008 ▶); program(s) used to refine structure: *SHELXL97* (Sheldrick, 2008 ▶); molecular graphics: *ORTEP-3* (Farrugia, 1997 ▶); software used to prepare material for publication: *SHELXL97*.

## Supplementary Material

Crystal structure: contains datablocks I, global. DOI: 10.1107/S1600536809014160/bt2921sup1.cif

Structure factors: contains datablocks I. DOI: 10.1107/S1600536809014160/bt2921Isup2.hkl

Additional supplementary materials:  crystallographic information; 3D view; checkCIF report

## Figures and Tables

**Table 1:** Hydrogen-bond geometry (Å, °)

*D*—H⋯*A*	*D*—H	H⋯*A*	*D*⋯*A*	*D*—H⋯*A*
O7—H7*C*⋯O2^i^	0.84	1.93	2.7475 (18)	163

Symmetry code: (i) 
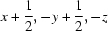
.

**Fig. 1.**
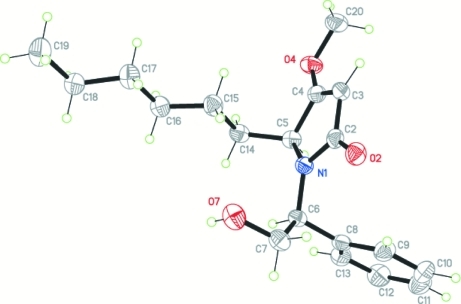
The molecular structure of the title compound with the atom-labeling scheme, showing 50% probability displacement ellipsoids. H atoms are drawn as spheres of arbitrary radius.

## supplementary crystallographic information

### Comment

Methyl tetramates bearing C-5 methyl substituents are key frameworks found in a number of bioactive natural products, such as dysideapyrrolidone and dolastatin (Royles, 1995). The title compound, (1), is one of the methyl tetramates which were synthesized when we researched the flexible method for the preparation of methyl (*S*)-5-alkyltetramate derivatives.

The title compound, (1), was obtained by the reaction of (3*S*,7a*R*)-7a-hexyl-7-methoxy-3-phenyl-2,3- dihydropyrrolo[2,1-*b*]oxazol-5(7a*H*)-one and triethylsilane using titanium (IV) chloride as catalyst. The absolute configuration (*S*) of the stereocentre C6 remains unchanged during the synthetic procedure. An X-ray crystal structure determination of the molecular structure of compound (1) was carried out to determine its conformation.

The phenyl and dihydropyrrolone rings form a dihedral angle of 83.8 (1)°. O—H···O hydrogen-bonding interactions lead to the formation of a chain parallel to the *a* axis.

### Experimental

The title compound was prepared by a method based on one described by Jiang *et al.* (2009). To a cooled (-78 °C) solution of (3*S*,7a*R*)-7a-hexyl-7-methoxy-3-phenyl-2,3-\ δihydropyrrolo[2,1-*b*]oxazol-5(7a*H*)-one (0.230 mmol) in dry dichloromethane (6 ml) was added dropwise a solution of TiCl_4_ (0.245 mmol), followed by Et_3_SiH (2.3 mmol) under nitrogen atmosphere. After being stirred at -78 °C for 2 h, the mixture was allowed to react at room temperature and stirred until the completion of the reaction. The mixture was quenched with saturated NaHCO_3_ solution. The organic layer was separated and the aqueous phase was extracted with CH_2_Cl_2_ (3 × 5 ml). The combined organic layers were washed with brine, dried over Na_2_SO_4_, and concentrated under reduced pressure. The residue was purified by flash chromatography to give (*S*)-5-hexyl-1-((*S*)- 2-hydroxy-1-phenylethyl)-4-methoxy-1*H*-pyrrol-2(5*H*)-one as colorless crystals. Single crystals were obtained by slow evaporation of a petroleum ether/ethyl acetate solution.

### Refinement

The hydrogen atoms were positioned geometrically (O—H = 0.84Å; C—H = 0.93, 0.98, 0.97 or 0.96Å for phenyl, tertiary, methylene or methyl H atoms respectively) and were included in the refinement in the riding model approximation. The displacement parameters of methyl and hydroxyl H atoms were set to 1.5*U*_eq_(C,O), while those of other H atoms were set to 1.2*U*_eq_(C). In the absence of significant anomalous scattering, Friedel pairs were merged; the absolute configuration was known from the synthesis.

### Crystal data

**Table:**

C_19_H_27_NO_3_	*F*(000) = 688
*M**_r_* = 317.42	*D*_x_ = 1.204 Mg m^−^^3^
Orthorhombic, *P*2_1_2_1_2_1_	Mo *K*α radiation, λ = 0.71073 Å
Hall symbol: P 2ac 2ab	Cell parameters from 9516 reflections
*a* = 9.6739 (17) Å	θ = 4.5–56.6°
*b* = 10.0995 (18) Å	µ = 0.08 mm^−^^1^
*c* = 17.929 (3) Å	*T* = 173 K
*V* = 1751.7 (5) Å^3^	Needle, colorless
*Z* = 4	0.56 × 0.32 × 0.23 mm

### Data collection

**Table:**

Bruker APEX CCD diffractometer	1773 independent reflections
Radiation source: fine-focus sealed tube	1732 reflections with *I* > 2σ(*I*)
graphite	*R*_int_ = 0.023
φ and ω scans	θ_max_ = 25.0°, θ_min_ = 2.3°
Absorption correction: multi-scan (*SADABS*; Bruker, 2001)	*h* = −11→11
*T*_min_ = 0.956, *T*_max_ = 0.982	*k* = −12→12
12545 measured reflections	*l* = −21→20

### Refinement

**Table:**

Refinement on *F*^2^	Primary atom site location: structure-invariant direct methods
Least-squares matrix: full	Secondary atom site location: difference Fourier map
*R*[*F*^2^ > 2σ(*F*^2^)] = 0.029	Hydrogen site location: inferred from neighbouring sites
*wR*(*F*^2^) = 0.079	H-atom parameters constrained
*S* = 1.13	*w* = 1/[σ^2^(*F*_o_^2^) + (0.0483*P*)^2^ + 0.1818*P*] where *P* = (*F*_o_^2^ + 2*F*_c_^2^)/3
1773 reflections	(Δ/σ)_max_ < 0.001
208 parameters	Δρ_max_ = 0.13 e Å^−^^3^
0 restraints	Δρ_min_ = −0.13 e Å^−^^3^

### Special details

**Table:**

Geometry. All e.s.d.'s (except the e.s.d. in the dihedral angle between two l.s. planes) are estimated using the full covariance matrix. The cell e.s.d.'s are taken into account individually in the estimation of e.s.d.'s in distances, angles and torsion angles; correlations between e.s.d.'s in cell parameters are only used when they are defined by crystal symmetry. An approximate (isotropic) treatment of cell e.s.d.'s is used for estimating e.s.d.'s involving l.s. planes.
Refinement. Refinement of *F*^2^ against ALL reflections. The weighted *R*-factor *wR* and goodness of fit *S* are based on *F*^2^, conventional *R*-factors *R* are based on *F*, with *F* set to zero for negative *F*^2^. The threshold expression of *F*^2^ > σ(*F*^2^) is used only for calculating *R*-factors(gt) *etc*. and is not relevant to the choice of reflections for refinement. *R*-factors based on *F*^2^ are statistically about twice as large as those based on *F*, and *R*- factors based on ALL data will be even larger.

### Fractional atomic coordinates and isotropic or equivalent isotropic displacement parameters (Å^2^)

**Table:**

	*x*	*y*	*z*	*U*_iso_*/*U*_eq_	
N1	0.23084 (14)	0.27270 (13)	0.08925 (7)	0.0264 (3)	
O2	0.01267 (12)	0.18490 (12)	0.08019 (6)	0.0339 (3)	
C2	0.09444 (17)	0.26555 (17)	0.10656 (9)	0.0275 (4)	
C3	0.06570 (18)	0.36770 (17)	0.16165 (9)	0.0314 (4)	
H3A	−0.0223	0.3883	0.1821	0.038*	
O4	0.21692 (13)	0.52314 (12)	0.22535 (7)	0.0381 (3)	
C4	0.18362 (19)	0.42683 (16)	0.17823 (9)	0.0300 (4)	
C5	0.30022 (18)	0.37368 (17)	0.13261 (9)	0.0294 (4)	
H5A	0.3699	0.3313	0.1662	0.035*	
C6	0.31243 (16)	0.17097 (16)	0.05156 (9)	0.0271 (4)	
H6A	0.4059	0.2102	0.0426	0.032*	
O7	0.23907 (13)	0.24369 (13)	−0.07171 (6)	0.0393 (3)	
H7C	0.3167	0.2780	−0.0799	0.059*	
C7	0.25486 (19)	0.13400 (18)	−0.02451 (8)	0.0324 (4)	
H7A	0.3177	0.0694	−0.0485	0.039*	
H7B	0.1639	0.0906	−0.0179	0.039*	
C8	0.33410 (18)	0.05271 (17)	0.10152 (9)	0.0293 (4)	
C9	0.2319 (2)	−0.04012 (17)	0.11356 (10)	0.0367 (4)	
H9A	0.1459	−0.0318	0.0884	0.044*	
C10	0.2528 (2)	−0.14481 (19)	0.16160 (11)	0.0441 (5)	
H10A	0.1815	−0.2081	0.1692	0.053*	
C11	0.3763 (2)	−0.1579 (2)	0.19852 (12)	0.0483 (5)	
H11A	0.3907	−0.2297	0.2318	0.058*	
C12	0.4785 (2)	−0.0666 (2)	0.18692 (11)	0.0460 (5)	
H12A	0.5640	−0.0751	0.2125	0.055*	
C13	0.45838 (19)	0.03788 (19)	0.13829 (9)	0.0360 (4)	
H13A	0.5307	0.0999	0.1301	0.043*	
C14	0.37058 (18)	0.47889 (17)	0.08519 (10)	0.0329 (4)	
H14A	0.4189	0.5416	0.1187	0.039*	
H14B	0.4415	0.4355	0.0538	0.039*	
C15	0.27425 (19)	0.55665 (18)	0.03513 (10)	0.0351 (4)	
H15A	0.2164	0.4941	0.0063	0.042*	
H15B	0.2120	0.6111	0.0664	0.042*	
C16	0.3508 (2)	0.64551 (19)	−0.01812 (11)	0.0401 (4)	
H16A	0.4100	0.7065	0.0109	0.048*	
H16B	0.4121	0.5905	−0.0497	0.048*	
C17	0.2578 (2)	0.7259 (2)	−0.06785 (10)	0.0421 (4)	
H17A	0.2078	0.7914	−0.0368	0.050*	
H17B	0.1881	0.6662	−0.0903	0.050*	
C18	0.3319 (2)	0.79856 (19)	−0.12980 (11)	0.0446 (5)	
H18A	0.4007	0.8594	−0.1075	0.054*	
H18B	0.3828	0.7334	−0.1606	0.054*	
C19	0.2366 (2)	0.8770 (2)	−0.17945 (11)	0.0494 (5)	
H19A	0.2908	0.9214	−0.2183	0.074*	
H19B	0.1877	0.9434	−0.1496	0.074*	
H19C	0.1695	0.8173	−0.2027	0.074*	
C20	0.1077 (2)	0.5705 (2)	0.27166 (10)	0.0457 (5)	
H20A	0.1427	0.6411	0.3041	0.069*	
H20B	0.0723	0.4977	0.3023	0.069*	
H20C	0.0330	0.6054	0.2404	0.069*	

### Atomic displacement parameters (Å^2^)

**Table:**

	*U*^11^	*U*^22^	*U*^33^	*U*^12^	*U*^13^	*U*^23^
N1	0.0263 (7)	0.0264 (7)	0.0263 (6)	−0.0003 (6)	0.0015 (5)	−0.0016 (6)
O2	0.0273 (6)	0.0347 (6)	0.0398 (6)	−0.0034 (6)	0.0012 (5)	0.0020 (5)
C2	0.0272 (8)	0.0288 (8)	0.0264 (7)	0.0012 (7)	−0.0001 (6)	0.0078 (7)
C3	0.0313 (8)	0.0318 (8)	0.0310 (8)	0.0057 (8)	0.0072 (7)	0.0056 (7)
O4	0.0436 (7)	0.0378 (7)	0.0329 (6)	0.0052 (6)	0.0017 (6)	−0.0096 (5)
C4	0.0388 (9)	0.0287 (8)	0.0224 (7)	0.0058 (7)	0.0008 (7)	0.0018 (7)
C5	0.0290 (8)	0.0308 (8)	0.0284 (8)	0.0025 (7)	−0.0025 (7)	−0.0024 (7)
C6	0.0243 (8)	0.0289 (8)	0.0281 (8)	0.0003 (7)	0.0019 (6)	−0.0037 (7)
O7	0.0343 (6)	0.0530 (8)	0.0307 (6)	0.0012 (6)	−0.0036 (5)	0.0045 (6)
C7	0.0314 (8)	0.0381 (9)	0.0275 (8)	−0.0015 (8)	0.0000 (7)	−0.0037 (7)
C8	0.0313 (8)	0.0287 (8)	0.0278 (8)	0.0034 (7)	0.0031 (7)	−0.0057 (7)
C9	0.0338 (9)	0.0325 (9)	0.0436 (9)	0.0001 (8)	−0.0015 (8)	−0.0013 (8)
C10	0.0493 (11)	0.0299 (9)	0.0532 (11)	−0.0018 (9)	0.0058 (10)	0.0034 (8)
C11	0.0597 (13)	0.0384 (10)	0.0468 (11)	0.0123 (10)	0.0018 (10)	0.0090 (9)
C12	0.0426 (11)	0.0520 (11)	0.0433 (10)	0.0109 (10)	−0.0065 (9)	0.0057 (10)
C13	0.0341 (9)	0.0378 (9)	0.0360 (8)	0.0014 (8)	−0.0007 (7)	−0.0018 (8)
C14	0.0284 (8)	0.0334 (9)	0.0370 (9)	−0.0021 (8)	0.0019 (7)	−0.0065 (7)
C15	0.0346 (9)	0.0349 (9)	0.0358 (8)	−0.0044 (8)	0.0029 (8)	−0.0008 (7)
C16	0.0397 (10)	0.0374 (10)	0.0433 (10)	−0.0016 (8)	0.0088 (8)	0.0020 (8)
C17	0.0441 (11)	0.0431 (10)	0.0390 (9)	−0.0063 (9)	0.0020 (9)	0.0034 (8)
C18	0.0523 (11)	0.0360 (9)	0.0455 (10)	0.0029 (9)	0.0133 (9)	0.0044 (9)
C19	0.0588 (13)	0.0482 (11)	0.0413 (10)	−0.0072 (11)	−0.0008 (10)	0.0069 (9)
C20	0.0594 (12)	0.0422 (10)	0.0355 (9)	0.0112 (10)	0.0099 (9)	−0.0067 (8)

### Geometric parameters (Å, °)

**Table:**

N1—C2	1.357 (2)	C11—H11A	0.9500
N1—C5	1.447 (2)	C12—C13	1.383 (3)
N1—C6	1.461 (2)	C12—H12A	0.9500
O2—C2	1.230 (2)	C13—H13A	0.9500
C2—C3	1.455 (2)	C14—C15	1.513 (3)
C3—C4	1.321 (3)	C14—H14A	0.9900
C3—H3A	0.9500	C14—H14B	0.9900
O4—C4	1.328 (2)	C15—C16	1.505 (2)
O4—C20	1.426 (2)	C15—H15A	0.9900
C4—C5	1.493 (2)	C15—H15B	0.9900
C5—C14	1.522 (2)	C16—C17	1.505 (3)
C5—H5A	1.0000	C16—H16A	0.9900
C6—C8	1.508 (2)	C16—H16B	0.9900
C6—C7	1.520 (2)	C17—C18	1.512 (3)
C6—H6A	1.0000	C17—H17A	0.9900
O7—C7	1.402 (2)	C17—H17B	0.9900
O7—H7C	0.8400	C18—C19	1.507 (3)
C7—H7A	0.9900	C18—H18A	0.9900
C7—H7B	0.9900	C18—H18B	0.9900
C8—C13	1.379 (2)	C19—H19A	0.9800
C8—C9	1.380 (2)	C19—H19B	0.9800
C9—C10	1.379 (3)	C19—H19C	0.9800
C9—H9A	0.9500	C20—H20A	0.9800
C10—C11	1.372 (3)	C20—H20B	0.9800
C10—H10A	0.9500	C20—H20C	0.9800
C11—C12	1.368 (3)		
			
C2—N1—C5	111.43 (14)	C13—C12—H12A	119.7
C2—N1—C6	126.40 (14)	C8—C13—C12	120.46 (18)
C5—N1—C6	119.56 (13)	C8—C13—H13A	119.8
O2—C2—N1	124.93 (16)	C12—C13—H13A	119.8
O2—C2—C3	127.41 (15)	C15—C14—C5	114.73 (14)
N1—C2—C3	107.65 (15)	C15—C14—H14A	108.6
C4—C3—C2	107.94 (15)	C5—C14—H14A	108.6
C4—C3—H3A	126.0	C15—C14—H14B	108.6
C2—C3—H3A	126.0	C5—C14—H14B	108.6
C4—O4—C20	115.87 (15)	H14A—C14—H14B	107.6
C3—C4—O4	133.12 (16)	C16—C15—C14	112.50 (15)
C3—C4—C5	111.50 (14)	C16—C15—H15A	109.1
O4—C4—C5	115.37 (15)	C14—C15—H15A	109.1
N1—C5—C4	101.38 (13)	C16—C15—H15B	109.1
N1—C5—C14	113.54 (13)	C14—C15—H15B	109.1
C4—C5—C14	113.14 (14)	H15A—C15—H15B	107.8
N1—C5—H5A	109.5	C17—C16—C15	113.79 (16)
C4—C5—H5A	109.5	C17—C16—H16A	108.8
C14—C5—H5A	109.5	C15—C16—H16A	108.8
N1—C6—C8	110.93 (12)	C17—C16—H16B	108.8
N1—C6—C7	112.95 (13)	C15—C16—H16B	108.8
C8—C6—C7	112.93 (13)	H16A—C16—H16B	107.7
N1—C6—H6A	106.5	C16—C17—C18	114.42 (17)
C8—C6—H6A	106.5	C16—C17—H17A	108.7
C7—C6—H6A	106.5	C18—C17—H17A	108.7
C7—O7—H7C	109.5	C16—C17—H17B	108.7
O7—C7—C6	112.79 (13)	C18—C17—H17B	108.7
O7—C7—H7A	109.0	H17A—C17—H17B	107.6
C6—C7—H7A	109.0	C19—C18—C17	113.52 (18)
O7—C7—H7B	109.0	C19—C18—H18A	108.9
C6—C7—H7B	109.0	C17—C18—H18A	108.9
H7A—C7—H7B	107.8	C19—C18—H18B	108.9
C13—C8—C9	118.44 (16)	C17—C18—H18B	108.9
C13—C8—C6	119.42 (16)	H18A—C18—H18B	107.7
C9—C8—C6	122.11 (16)	C18—C19—H19A	109.5
C10—C9—C8	120.91 (18)	C18—C19—H19B	109.5
C10—C9—H9A	119.5	H19A—C19—H19B	109.5
C8—C9—H9A	119.5	C18—C19—H19C	109.5
C11—C10—C9	120.20 (19)	H19A—C19—H19C	109.5
C11—C10—H10A	119.9	H19B—C19—H19C	109.5
C9—C10—H10A	119.9	O4—C20—H20A	109.5
C12—C11—C10	119.41 (18)	O4—C20—H20B	109.5
C12—C11—H11A	120.3	H20A—C20—H20B	109.5
C10—C11—H11A	120.3	O4—C20—H20C	109.5
C11—C12—C13	120.57 (19)	H20A—C20—H20C	109.5
C11—C12—H12A	119.7	H20B—C20—H20C	109.5
			
C5—N1—C2—O2	−176.98 (14)	C5—N1—C6—C7	−142.17 (14)
C6—N1—C2—O2	−15.5 (3)	N1—C6—C7—O7	55.07 (19)
C5—N1—C2—C3	2.15 (18)	C8—C6—C7—O7	−178.04 (14)
C6—N1—C2—C3	163.58 (13)	N1—C6—C8—C13	−100.67 (18)
O2—C2—C3—C4	175.95 (16)	C7—C6—C8—C13	131.38 (16)
N1—C2—C3—C4	−3.15 (18)	N1—C6—C8—C9	77.47 (18)
C2—C3—C4—O4	−178.40 (17)	C7—C6—C8—C9	−50.5 (2)
C2—C3—C4—C5	2.92 (19)	C13—C8—C9—C10	0.5 (3)
C20—O4—C4—C3	4.1 (3)	C6—C8—C9—C10	−177.68 (16)
C20—O4—C4—C5	−177.24 (15)	C8—C9—C10—C11	0.2 (3)
C2—N1—C5—C4	−0.46 (17)	C9—C10—C11—C12	−0.3 (3)
C6—N1—C5—C4	−163.32 (13)	C10—C11—C12—C13	−0.2 (3)
C2—N1—C5—C14	−122.12 (15)	C9—C8—C13—C12	−1.0 (3)
C6—N1—C5—C14	75.02 (18)	C6—C8—C13—C12	177.17 (16)
C3—C4—C5—N1	−1.61 (18)	C11—C12—C13—C8	0.9 (3)
O4—C4—C5—N1	179.46 (13)	N1—C5—C14—C15	60.86 (19)
C3—C4—C5—C14	120.33 (16)	C4—C5—C14—C15	−53.98 (19)
O4—C4—C5—C14	−58.60 (19)	C5—C14—C15—C16	−171.94 (15)
C2—N1—C6—C8	−70.19 (19)	C14—C15—C16—C17	−179.05 (15)
C5—N1—C6—C8	89.88 (17)	C15—C16—C17—C18	−170.57 (17)
C2—N1—C6—C7	57.8 (2)	C16—C17—C18—C19	179.28 (17)

### Hydrogen-bond geometry (Å, °)

**Table:**

*D*—H···*A*	*D*—H	H···*A*	*D*···*A*	*D*—H···*A*
O7—H7C···O2^i^	0.84	1.93	2.7475 (18)	163

Symmetry codes: (i) *x*+1/2, −*y*+1/2, −*z*.
